# A Case of Type V Hyperlipoproteinemia Resistant to Insulin Treatment

**DOI:** 10.7759/cureus.41424

**Published:** 2023-07-05

**Authors:** Shaunak Mangeshkar, Natalia Nazarenko, Dimitrios Varrias, Michail Spanos, Pawel Borkowski, Majd Al Deen Alhuarrat, Weijia Li, Preeti Kishore, Robert T Faillace

**Affiliations:** 1 Internal Medicine, Albert Einstein College of Medicine, Jacobi Medical Center, New York, USA; 2 Cardiovascular Medicine, Massachusetts General Hospital, Harvard Medical School, Boston, USA; 3 Cardiology, AdventHealth Orlando, Orlando, USA; 4 Medicine, Jacobi Medical Center, New York, USA

**Keywords:** insulin resistance, cardiovascular disease, hypertriglyceridemia induced pancreatitis, severe hypertriglyceridemia, multifactorial chylomicronemia syndrome

## Abstract

Type V hyperlipoproteinemia or multifactorial chylomicronemia syndrome is a rare lipid disorder triggered mainly by uncontrolled diabetes, obesity, poor diet, or particular medications. It is associated with an increased risk of acute pancreatitis and accelerated coronary artery disease which may manifest in younger age groups. We present a case of a 42-year-old male who presented to the emergency department (ED) complaining of a non-healing hand injury. Upon laboratory workup, the patient was found to have an elevated total cholesterol (TC) of 1129 mg/dL, very low levels of high-density lipoprotein (HDL) and triglycerides (TG) > 4000 mg/dL with an inability to calculate low-density lipoprotein (LDL). Lipoprotein electrophoresis revealed an actual TG level of > 7000 mg/dL, increased chylomicrons, normal B and pre-B-lipoproteins, and increased L-lipoproteins with an elevated Apolipoprotein B. Despite these derangements, the patient did not exhibit any abdominal complaints, demonstrating a normal lipase level. The physical exam was indicative of bilateral arcus senilis and obesity. Insulin drip was initiated along with intravenous (IV) hydration and it required 12 days to bring triglycerides down to less than 1000 mg/dL. The total cholesterol was also seen to be down trending to around 500 mg/dL and the HDL improved to 22 mg/dL. We present this case as a unique presentation of asymptomatic chylomicronemia resistant to insulin treatment with an elevated ApoB but with no evidence of pancreatitis or coronary artery disease.

## Introduction

Multifactorial chylomicronemia syndrome (MCS), previously named type V hyperlipoproteinemia, is a rare polygenic disorder characterized by the accumulation of triglycerides, chylomicrons, and very low-density lipoproteins (VLDL) in plasma, which appears to be the most common reason for hypertriglyceridemia with estimated accounting prevalence of 1:250 to 1:600 in the North American population [1]. A definition of hyperchylomicronemia is reflected in triglyceride (TG) level > 2000 mg/dL due to the fact that chylomicrons are detected in plasma after TG reaches level >1000 mg/dL, although caution should be taken as patients might have limited or no symptoms even with TG level 20,000-30,000 mg/dL. High TG values might mislead other laboratory results and falsely lower sodium, hemoglobin, and amylase, therefore, a diagnosis of acute pancreatitis can be missed [2].

This disorder manifests as late-onset chylomicronemia and, in a majority of cases, is triggered by risk factors including but not limited to obesity, uncontrolled diabetes mellitus type 1 and 2, metabolic syndrome, alcohol consumption, non-adherence to a healthy diet, administration of estrogens and testosterone, usage of beta-blockers, diuretics, selective serotonin reuptake inhibitors (SSRIs), HIV medications - protease inhibitors, pregnancy state, isotretinoin, multiple myeloma, systemic lupus erythematosus (SLE) and malignant lymphoma [2,3]. As the literature shows, it is a disorder with a rare familial occurrence and unclear relationship with accelerated coronary artery disease (CAD) [2], although it has a known association with acute pancreatitis [1]. Although inherited familial type I hyperlipoproteinemia secondary to lipoprotein lipase deficiency which also manifests in younger age has a defined association with accelerated coronary artery disease (CAD), the question of whether hypertriglyceridemia associated with MCS accelerates CAD remains unanswered [4].

## Case presentation

**Figure 1:**
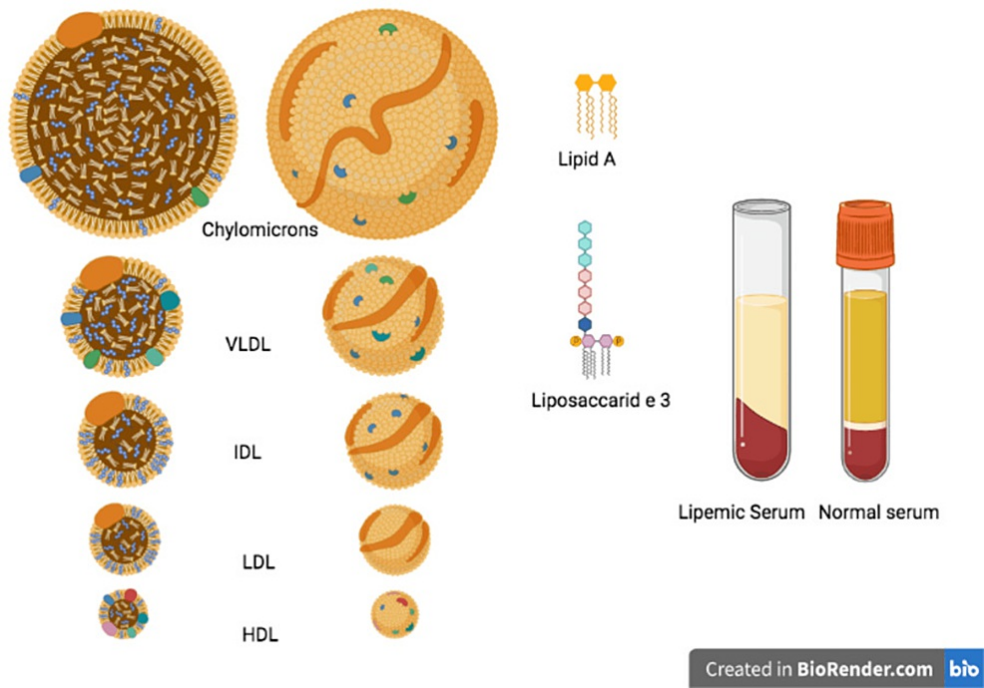
Lipemic Plasma Image Credits: Dimitrios Varrias. IDL: Intermediate-density lipoprotein; VLDL: Very low-density lipoprotein; HDL: High-density lipoprotein; LDL: Low-density lipoprotein.

A 42-year-old male, originally from Jamaica with a past medical history of uncontrolled diabetes with HbA1c 16.3% presented to the emergency department with a hand injury. A basic laboratory workup was done. Due to lipemic plasma seen during blood draws (Figure 1), a lipid profile was ordered, notable for the following lipid abnormalities: low high-density lipoprotein (HDL) cholesterol, extremely elevated triglycerides and total cholesterol. Furthermore, a blood glucose level was very high along with an increased anion gap, blood gases represented normal pH 7.37 (normal range 7.32-7.43), sodium level was low (Table 1), likely representing pseudo hyponatremia. Lipoprotein electrophoresis showed total cholesterol > 700 mg/dL with triglyceride of 7344 mg/dL, increased chylomicrons and decreased A-lipoproteins. Hypothyroidism was ruled out with thyroid stimulating hormone (TSH) in the normal range of 1.02 (normal range: 0.670-6.900 U/L). Apolipoprotein B was found to be elevated while alanine transaminase (ALT) and aspartate transaminase (AST) were within normal ranges of 44 and 5 U/L, respectively. Physical examination was notable for bilateral arcus senilis and a body mass index (BMI) of 30. No eruptive xanthomas were noted.

**Table 1:** Initial metabolic parameters and trend of lipid levels over the course of hospitalization. HDL: High-density lipoprotein

	Normal value	Admission / Day 1	Day 4	Day 6	Day 11	Day 15
HDL Cholesterol (mg/dL)	27 - 67	< 3	10	14	18	22
Total Cholesterol (mg/dL)	120 - 220	> 1112	863	856	677	523
Triglycerides (mg/dL)	40 - 160	7344	3398	2160	1157	768
Chylomicrons	Normal	Increased	-	-	-	-
Apolipoprotein B (mg/dL)	< 60	153	-	-	-	-
Non-HDL Cholesterol (mg/dL)	Unable to calculate	Unable to calculate	1014	-	-	-
Blood sugar (mg/dL)	70 - 126	491	-	-	-	-
HbA1C, %	< 6.5	16.8	-	-	-	-
Sodium (mg/dl)	125 - 135	121	-	-	-	-

**Figure 2:**
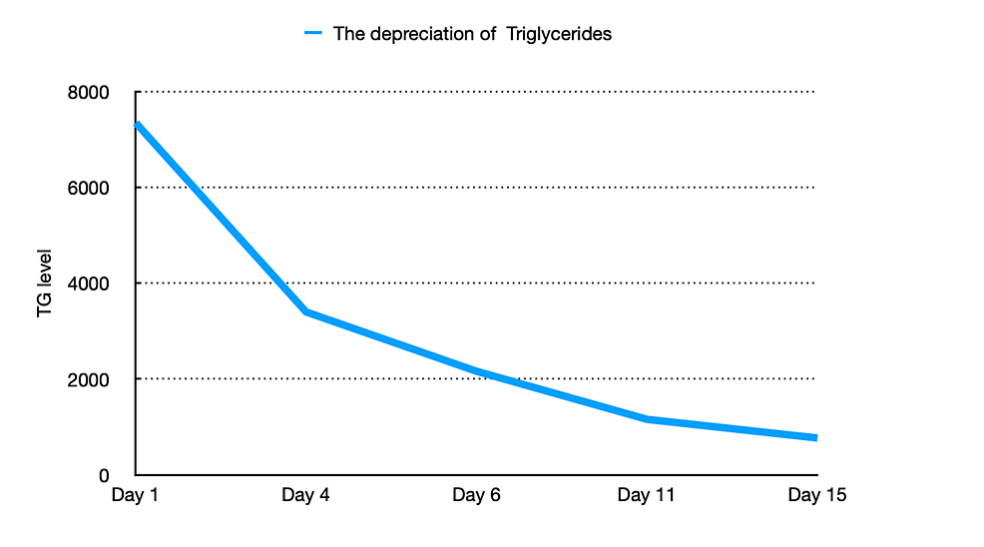
Trend in triglyceride levels over the course of admission

The patient was started on an insulin infusion with a blood glucose goal of 140-180 mg/dL. 5% Dextrose in water was not given in order to prevent lipogenesis in the liver, therefore the patient was receiving normal saline instead. The patient was not allowed anything by mouth and despite mentioned efforts, he remained consistently hypertriglyceridemic with achieved TG reduction below 50% from admission by day 4. Additionally, he was started on fenofibrate 134 mg daily, atorvastatin 80 mg daily, and omega-3-ethyl esters 2 grams daily. After 15 days of treatment, TG had considerably lowered (Figure 2, Table 1), when the patient was successfully transitioned to long-acting insulin glargine of eight units daily. A fat-free diet was introduced, and treatment with Metformin was resumed. The patient remained asymptomatic throughout the hospital course without any associated cardiac or abdominal pain. The patient was discharged on fenofibrate, atorvastatin, liraglutide, and insulin glargine eight units daily.

During outpatient follow-up visits over the ensuing six months after discharge, his medications were switched to include a maximum dose of metformin, and insulin was stopped. The patient stated full compliance to prescribed medications and continued to remain asymptomatic.

## Discussion

The chylomicronemia syndrome is an entity defined by the presence of intermittent or persistent fasting chylomicronemia leading to extreme elevations in triglyceride levels. Traditionally, severe hypertriglyceridemia has been defined as serum triglyceride levels > 500 mg/dL [5]. The three etiologies of the chylomicronemia syndrome are Multifactorial Chylomicronemia Syndrome (MCS), Familial Chylomicronemia Syndrome (FCS), and Familial Partial Lipodystrophy (FPLD) [6]. Multifactorial chylomicronemia syndrome (MCS), polygenic late-onset chylomicronemia, or Type V hyperlipoproteinemia, has been identified as the most common form of the above syndrome [1].

The genetics involving the development of multifactorial chylomicronemia syndrome is centered around either of the following two mechanisms: polygenic - accumulation of minor genetic variations called single nucleotide polymorphisms (SNPs), each contributing to some extent to the development of hypertriglyceridemia or presence of a single aberrant genetic variation in one of the five canonical genes regulating triglyceride metabolism namely LPL, APOC2, APOA5, GPHIBP1, and LMF1 [1,7]. In a study involving > 500 Caucasian patients with severe hypertriglyceridemia, a high polygenic risk score, calculated by quantifying SNPs extracted from across the genome, was the most common genetic determinant [8]. However, in most patients developing chylomicronemia syndrome, there is a need for the concomitant presence of secondary non-genetic factors, which additionally contribute to the worsening of hypertriglyceridemia. These predominantly include medical conditions such as poorly controlled diabetes, obesity, kidney disease, excessive alcohol use, and hypothyroidism, as well as medications such as diuretics, estrogen, corticosteroids, certain immunomodulatory drugs, and protease inhibitors [7]. Poorly controlled diabetes, as seen in this case, contributes to hypertriglyceridemia by increasing the conversion of free fatty acids released from adipose tissue to triglycerides and by stimulating de novo lipogenesis in the liver alongside increased production of triglyceride-rich lipoproteins [9].

The mechanism of development of hypertriglyceridemia in chylomicronemia syndrome has been postulated to be a combination of overproduction and reduced clearance of triglycerides. Endogenous triglycerides are produced and secreted by the liver continuously through very low-density lipoproteins (VLDL), while triglycerides in dietary fat are incorporated into chylomicrons when absorbed in the intestine. The enzyme lipoprotein lipase (LPL), synthesized in adipose tissue and skeletal muscle capillaries, is responsible for triglyceride conversion to fatty acids and subsequent clearance from the blood and constitutes the primary mechanism of triglyceride metabolism. The function of LPL is influenced by several apolipoproteins, including but not limited to Apo CII, which activates LPL, and Apo CIII, which inhibits LPL activity, Glycosylphosphatidylinositol-anchored high-density lipoprotein-binding protein 1 (GPIHBP1), which transports LPL to the endothelial surface to exert its action [6]. When the triglyceride levels in serum exceed 500-700 mg/dL, the clearance mechanisms become saturated such that further intake of triglycerides tends to increase the level of triglyceride-rich VLDL and chylomicrons, which may be detected in the plasma even after an overnight fast, thereby leading to the onset of chylomicronemia syndrome.

Acute pancreatitis is the most common clinical manifestation seen in patients with chylomicronemia syndrome. The relative risk of developing pancreatitis in this subset of patients is at least seven times higher than that of the general population [10-12]. The risk increases with triglyceride levels > 500 mg/dL and is markedly elevated at >1000 mg/dL. Interestingly, our patient did not develop pancreatitis and was only incidentally diagnosed with hyperlipidemia despite extreme triglyceride levels elevations (>7000 mg/dL). The pathogenesis of pancreatitis is believed to be related to the release of pro-inflammatory lysolecithin and fatty acids produced by the hydrolysis of triglycerides in pancreatic capillaries [7]. Furthermore, a meta-analysis comparing outcomes in severe hypertriglyceridemia (HTG)-induced pancreatitis vs non-HTG-induced pancreatitis revealed significantly worse outcome measures for patients with HTG-induced pancreatitis, including higher odds of shock and mortality [13]. In an attempt to further stratify the clinical phenotypes in patients with multifactorial chylomicronemia syndrome, a study was performed that compared patients with the presence or absence of a rare genetic variation in the five canonical triglyceride genes mentioned earlier. The study revealed that, in patients with genetic variation, there was a heightened risk of acute pancreatitis and multiple episodes of pancreatitis [14]. Clinically these patients may present with upper abdominal pain classically associated with pancreatitis. Additionally, physical exam findings seen in patients with chylomicronemia syndrome include eruptive xanthomas and corneal arcus, as seen in our patient, which is attributable to elevated lipid levels.

The risk of cardiovascular disease (CVD) is also elevated in patients with chylomicronemia syndrome. This has been deemed to be due to the deposition of atherogenic cholesterol molecules in the walls of blood vessels by triglyceride-rich lipoprotein remnants and not by chylomicron particles directly, as they are too large to penetrate vascular walls [10]. Notably, some studies have shown that the incidence of CVD may not correlate directly with the magnitude of hypertriglyceridemia [15]. Reports suggest an increased prevalence of non-alcoholic fatty liver disease (NAFLD) in patients with increased circulating triglyceride levels. This has been associated with the eventual progression to non-alcoholic steatohepatitis (NASH) and cirrhosis, as well as the development of insulin resistance and metabolic syndrome in patients with chylomicronemia [9].

The primary goal of treatment in patients with chylomicronemia syndrome is to reduce the serum levels of triglycerides and consequently decrease the risk of acute pancreatitis. A target serum triglyceride level < 500 mg/dL is sufficient to reduce the risk of acute pancreatitis considerably [16,17]. Other goals of treatment include decreasing the risk of cardiovascular disease. Of note, it is essential to distinguish between the etiologies of chylomicronemia syndrome since the treatment approach varies significantly [6].

In general terms, treatment should involve dietary fat restriction for all patients tolerating oral intake, until triglyceride levels are lowered below 1000 mg/dL [6]. For those developing acute pancreatitis, adequate fluid resuscitation and pain control should be ensured. More specifically, treatment options include plasmapheresis or patients admitted to the inpatient setting with hypertriglyceridemia-induced pancreatitis, especially in the presence of features concerning for multiple organ dysfunction. Intravenous insulin infusion can be used as a therapeutic measure in patients with poor prognostic factors, where plasmapheresis cannot be performed and in those with concomitant hyperglycemia. We preferred to start IV insulin due to poorly controlled diabetes with severe hyperglycemia. The patient needed 12 days of IV insulin drip before treatment was eventually transitioned to long-acting insulin. This could potentially be explained by a very high degree of insulin resistance complicating his uncontrolled diabetes. With regards to outpatient management in patients with Multifactorial Chylomicronemia Syndrome (MCS), given the major role played by secondary modifiable factors such as diabetes, chronic kidney disease, alcohol use, and drugs like estrogens, diuretics, beta-blockers in the pathogenesis of the disease, addressing and controlling them constitutes an integral part of the treatment [1]. To that effect, optimal outpatient diabetes control is a very critical measure. Switching therapeutic drug classes to lipid-neutral categories and alcohol cessation can be practical tools to prevent the worsening of hypertriglyceridemia. Low-fat and low-carbohydrate diets, especially when monitored by specialized dieticians, have been shown to significantly decrease triglyceride levels by reducing the formation of triglyceride-rich chylomicrons and endogenous VLDL, respectively [18]. Fibrates can be used as pharmacotherapy in conjunction with non-pharmacologic measures to reduce triglyceride levels [7] further. High-dose omega-3 fatty acids and Niacin can be added to augment the lipid-lowering effect, although Niacin can worsen glycemic control [7,19,20]. In terms of reducing cardiovascular risk, statins are considered the mainstay of therapy.

Finally, new emerging therapies for hypertriglyceridemia have shown promising results. These include medications targeting Apo CIII and angiopoietin-like protein (ANGPTL), amongst others [21,22]. ANGPTL3, 4 and 8 have been implicated in lipoprotein metabolism due to their action, namely inhibition of LPL and endothelial lipase [23]. An inhibitor of ANGPTL3 - Evinacumab has been approved in the US since 2021 as an adjunct cholesterol-lowering modality [24]. Further, anti-sense oligonucleotides namely Volanesorsen targeting ApoCIII mRNA and Vupanorsen targeting hepatic ANGPTL3 mRNA have also shown efficacy in terms of reduction of circulating triglyceride levels and episodes of pancreatitis in some clinical trials [22].

## Conclusions

In conclusion, our case report highlights the clinical course of a patient with severe hypertriglyceridemia, eventually diagnosed with multifactorial chylomicronemia syndrome. We further emphasize that although patients can be asymptomatic even with extreme elevations in triglyceride levels, treating physicians should be aware of the potential complications of this condition. Treatment of this syndrome is directed towards decreasing the risk of acute pancreatitis and cardiovascular disease and should include a well-rounded approach to managing secondary conditions in addition to dietary and pharmacological measures.
